# Technological knowledge progress: Were famous laws almost correct in developing and emerging economies?

**DOI:** 10.1371/journal.pone.0283107

**Published:** 2023-05-08

**Authors:** Voxi Heinrich Amavilah, Antonio Rodriguez Andres

**Affiliations:** 1 Economics/Division of Behavioral and Social Sciences, Estrella Mountain College, Avondale, Arizona, United States of America; 2 Department of Economics, Faculty of Management and Technology, German University in Cairo (GUC), New Cairo, Egypt; Al-Zaytoonah University for Science and Technology, STATE OF PALESTINE

## Abstract

Do the famous laws of the motion of technological progress like the Moore’s Law, Wright’s Law, Goddard’s Law, and their derivatives explain the technological knowledge progress of developing and emerging economies? The aim of this paper is to investigate that question. For that purpose, we rationalize an existing framework (Nagy et al. 2013) and employ it on a panel data set of 66 developing and emerging market economies over the 1995–2017 period. Empirical evidence is mixed. Some of the results confirm a positive relationship between technological knowledge progress and the progress of time. Other instances indicate that the slow rate of learning delays the doubling time by 18 years. Yet other results predict that this group of countries will double its rate of progress in 4–5 years. The explanatory power varies across the laws, with most laws suggesting acceptance of the hypotheses that the included variables affect the technological knowledge progress while others recommending that we “do not accept” the hypothesis that *in-situ* scale and hence cumulative GDP per capita explain the technological knowledge progress of these countries. Practical policy implications, which this group of countries can use to assess and address constraints to the technological knowledge progress, are also discussed.

## 1. Introduction

This paper investigates whether some well-known laws of the motion of technological progress explain the progress of technological knowledge. The goal is to assess the practical utility for predicting technological knowledge progress in a cross-section panel of 66 developing and emerging market economies (henceforth, **DEMEs**) over the 1995–2017 period. The topic is relevant for understanding the role of technology and technological change in the progress of this group of countries. The importance of the famous laws of motion of technological progress has long been acknowledged in many applications at the microeconomic level in some industries and mostly industrialized economies. How long? [1] provides one of the early written accounts of the importance of technological change in production, distribution, and consumption. Although [2] describes what we call *Hero’s effects of technology* [3], technology is a set of survival strategies that are constantly changing with human needs and improvements in knowledge [4, 5]. A delayed Hero’s technology effect refers to situations when the effects of new inventions and innovations are zero or inappreciably minimal in their first year(s). Many inventions by Hero of Alexandria were viewed this way before they took off. One can argue that the spear evolved into a bow and arrow as a technology for hunting and warfare. When game became scarce and hard to catch, and warfare difficult to win with bow and arrow, the gun followed naturally for war and to defend agriculture as an institutional technology. With some of the basic needs met, the search for varieties of freedom began [6, 7], and it, too, needed technology. It makes sense then that [8] writes about “technology and social change, technology and aging, technology, and war and peace,” whereas according to [9] technology and organization gave birth to capitalism [10]. Consequently, although absent from Adam Smith’s [11] *Wealth of Nations*, technological change is the source of the wealth of nations [12–14], which in turn underscores the endogenous interactions between technology and society [15, 16]. The aggregate “wealth of nations” that Smith wrote about is inconceivable without a critical mass of the “scientific wealth of nations” [17].

Clearly, at the microeconomic level research and teaching on technology and technological progress have advanced greatly [18, 19]. While they have focused on the industrialized countries mainly, such efforts have also laid a strong foundation for the transfer and diffusion of technological knowledge to developing and emerging economies despite the potential for Jones’s [20] paradoxes. Jones’s first paradox: “A foreign technological advance in the production of a commodity not produced at home may worsen the home country’s real income.” Jones’s second paradox: “A foreign technological advance in a commodity the home country exports may serve to raise home real incomes” [20] (p. 1). Such processes are inevitable because the importance of technology and technological change is also now an inevitable part of economic growth modeling at the macroeconomic level [21–26]. However, there remains a gap in the understanding of the evolution and importance of technological knowledge progress in the DEMEs. Even though technological knowledge plays an increasingly crucial part, in this group of countries the dearth of data hampers studies like this one and therefore the lack of study handicaps both policy and future research. This is the problem we address.

Recently a new and growing generation of research has returned to this subject. For example [27], have attempted to explain cumulative technological knowledge by characterizing the structure of the knowledge base first to argue that the base and its “cumulativeness” are proportionally related. Hence, economies with large knowledge bases cumulate more technological knowledge and grow faster than their counterparts. Using European countries as an example [28], show that different countries occupy different knowledge “spaces”, and their knowledge spaces explain their resilience. Hence, economies “endowed with technologically coherent capabilities adapt better in times of economic downturn, and that resilience is influenced by a region’s capacity to generate new growth paths” (p. 2). Consequently, economies have different breadths and depths of technological knowledge, so that the relationships among the production of knowledge, technological knowledge, and cumulative technological knowledge are multidimensional and dynamic. Where past technological knowledge has been shallow, production scale is low, knowledge accumulation slow, and the rate of current technological progress is even slower [29, 30]. These dimensions (breadths and depths) do not depend on technical factors alone, but also on a wide range of other influences as carefully studied by [31], [32], and [33]’s review of [31] and [32].

In a recent article [34], find six waves of technology diffusion in the data spanning 1870–2019 for 104 countries. Their analysis reveals differential impacts of technology diffusion on education at different levels, with such impacts favoring industrialized economies and leaving non-industrialize country with a lot of catching up to do. Similar differences were observed by [35] in the African context. Despite all this, *a gap exists in our understanding of the laws of motion of technological knowledge progress in the DEMEs*. In fact, [henceforth, NFBT, 36] have argued that “*there are many hypotheses about technological progress*” but that it remains unclear if “*any*” are “*good*” (p. 1, emphasis added). NFBT then proceed to assess six well-established statistical laws of technological progress: Moore’s Law [37], Wright’s Law [38], Goddard’s Law [39], Nordhaus’s Law [40], and Sinclair, Klepper, and Cohen’s Law [SKC, 41]. From [36] we ask: Do the famous laws of the motion explain the technological progress of DEMEs?

Again, the research question is important to ask and pursue because there remains a gap in the applied literature between what is known of the laws of motion of technological progress at the engineering microeconomic level as opposed to the macroeconomic level. In fact, from that literature has sprouted a new and growing literature in the areas of industrial engineering and management for quality operations [42], knowledge management improvement [43], social supply chain and sustainable performance and knowledge management improvement [44], technology knowledge progress [45], and even circular economy and its dynamics [45, 46]. Quite obviously these are important, interesting, and represent fruitful areas for future research. However, these strands of literature are afield. We are interested in the practical experience of countries at the macroeconomic level with DEMEs as our unit of analysis. We address the gap by reframing, modifying, and estimating the laws for a group of 66 developing and emerging economies over the 1995–2017 period. We make two contributions to extant literature. First, as far as we are aware, this is the first of its kind application of these models to DEMEs. One may argue that this contribution is not s*ufficient*, but it is indeed *necessary* if both policy and research were to move this group of countries toward their own and the world technological frontiers. The fact that the results are show that not all models fit the previous example cautions general statements about technological progress: there is no such thing as one size fits all, and this group of countries present special consideration in that sense. A second contribution is the finding that in-situ production scale in this group of countries is too small to allow for technological change to cut cost sufficiently enough to allow for the fast accumulation of technological knowledge and speed up the catch-up time, which is consistent with [47], [48], and [49] as we outline later. Both contributions have important implications for policy and future research.

In the next section we provide a methodological as well as empirical structure for our objective, and estimate the laws separately and in combinations. Section 3 presents and discusses the results, while Section 4 concludes the paper.

## 2. Famous laws of technological progress: A production function framework

Economic theory predicts a clear relationship between human progress measured by economic activity (*Y*_*i*_(*t*)), technological knowledge (*A*_*i*_(*t*)), and productive resources (*X*_*i*_(*t*)). For example, in one version of economic growth theory, as in [21–23] and others, the relationship is unidirectional with causation assumed to come from *A*_*i*_(*t*) to *Y*_*i*_(*t*), i.e., *Y*_*it*_ = *A*_*i*_(*t*)*f*(*X*_*i*_(*t*)), and *A*_*i*_(*t*) is exogenous. The endogenous growth theory treats *Y*_*i*_(*t*) and *A*_*i*_(*t*) as jointly determined such that,

$$Y_{i}(t)={f[A}_{i}(t)X_{i}(t)],\;i=1,2,3,\ldots ,N=66\;countries,t=1,2,3,\ldots ,T=23\;years.$$ (1)

Dividing both sides of (1) by some *X*_*j*_(*t*), *i* ≠ *j*, gives *y*_*i*_(*t*) per *X*_*j*_(*t*) as in

$$y_{i}(t)={f[A}_{i}(t)(x_{i}(t)],{y_{it}=\frac{Y_{i}(t)}{X_{j}(t)},x}_{i}(t)=\frac{X_{i}(t)}{X_{j}(t)}).$$ (2)

Solving (2) for *A*_*i*_(*t*) we get

$$A_{i}(t)={f(y}_{i}(t)x_{i}^{-1}(t)).$$ (3)

Economists have imposed different functional forms on (2), with the most common of these being the multiplicative Cobb-Douglas function, which gives *A*_*i*_(*t*) in (3) as an nth root of the output-input ratio, (*y*_*i*_(*t*)/*x*_*i*_(*t*)). In this paper we follow NFBT and impose the familiar laws of Moore, Wright, Goddard, and combined modifications. For example, a combination of [37] and [38] gives us:

$$A_{i}(t)=(Wright^{\prime }s\;Law)(Moore^{\prime }s\;Law)e^{error_{i}(t)}=A_{0}(t)y_{i}^{-w}(t)e^{-mt+\varepsilon_{i}(t)},$$ (4)

where *A*_0_>0, *w*>0, and *m* > 0 are the Wright and Moore coefficients, respectively. NFBT present another formulation which combines [37] and [38] into

$$A_{i}(t)=(Wright^{\prime }s\;Law)(Goddard^{\prime }s\;Law)e^{error_{i}(t)}=A_{0}(t)y_{i}^{-w}(t){ỹ}_{i}^{-g}(t)e^{\varepsilon_{i}(t)}.$$ (5)

Moreover, combining the [39] and [37] results in:

$$A_{i}(t)={(Goddard^{\prime }s\;Law)(Moore^{\prime }s\;Law)e^{error_{i}(t)}=A}_{0}(t){ỹ}_{i}^{-g}(t)e^{-mt+\varepsilon_{i}(t)},$$ (6)

where in (5) and (6) *y*_*i*_ is current (*in-situ*) scale which is GDP per capita, and ${ỹ}_{i}(t)=\sum_{i}^{n}y_{i}(t)$ is cumulative production the rate of which is the learning rate. Finally, we generalize (4)–(6) to,

$$A_{it}=(Nordhaus^{\prime }s\;Law)(SKC^{\prime }s\;Law)e^{error_{i}(t)}{=A}_{0}(t)y_{i}^{-w}(t){ỹ}_{i}^{-g}(t)e^{mt+\varepsilon_{i}(t)}.$$ (7)

As for interpretation the original laws (4)–(6) represent real unit cost such that the LHS is the inverse of the RHS; the problem there is cost minimization. Here we assume the maximization of technological knowledge progress. This is not a wild assumption at all as from both [47] and [48] technological improvements tend to reduce costs and thereby increase productivity. In fact [49], shows that technical change and productivity are closely related [50, 51]. Thus, often *A*_*i*_(*t*) is said to be total factor productivity and this is the reason to decompose productivity as called for by [52] and [53]. Thus, the progress of (3) is consistent with standard economic theory. [54], for example, illustrates that the progress of technological knowledge depends on two broad “driving forces”: External (exogenous) driving forces and internal (endogenous) driving forces. [55] has dubbed all external forces the “superstructure” and internal forces form the “infrastructure.” Among the latter are resources, logical reasoning, creativity, and both reinforcing and impeding responses to change. External forces can be social, political, and or ideological [56]. [57] has suggested that each epoch deals with its own changes brought about by internal social demands. According to [18], a key determinant of technological change is resources. Resources produce output for a gain (profit, wage, rent, and interest at the microeconomic level or just income/output at the macroeconomic level). Under free market conditions demand and supply determine the levels of input and output. As we restate more precisely later, our formulation accommodates that logic and permits our estimations of *A*_*i*_(*t*).

## 3. Data and methodology

This section is structured in the following way: First, we describe the data employed in the analysis, and then the empirical model and estimation strategy.

### 3.1 Data

**Fig 1:**
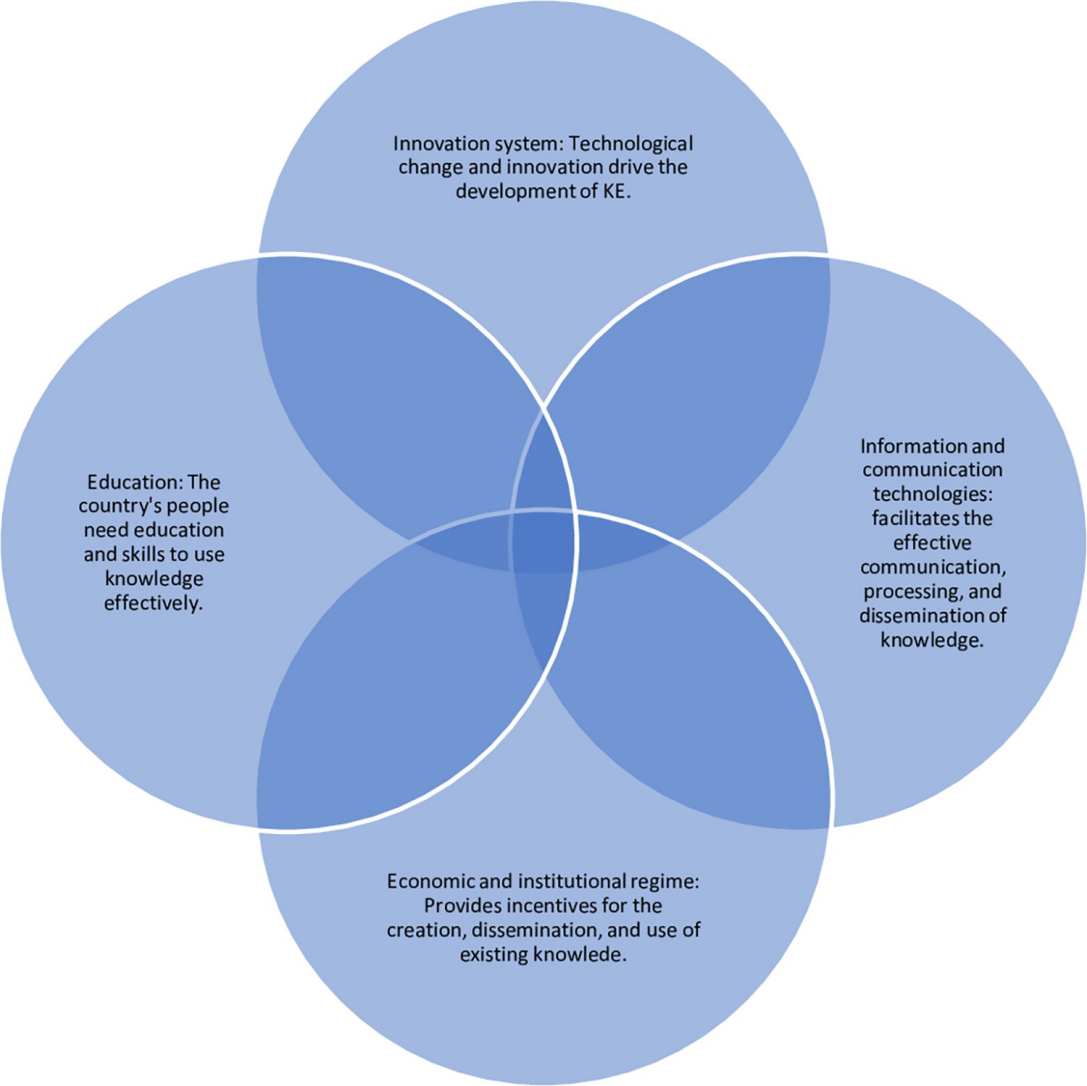
KEI pillars. Source: own creation.

The 66 DEMEs are covered in Table 1 and the selection was dictated by the data availability. For the dependent variable *A*_*i*_(*t*) we use a Knowledge Economy Index (KEI). The data for this variable derive from the [58, 59] updated using deep learning neural artificial networks. As Fig 1 shows, primary, secondary, and tertiary enrollment ratios represent the education pillar. Fixed broadband, telephone lines, and internet subscription indicate the information and communication technologies pillar. The innovation pillar reflects innovation outputs measured as scientific articles and patent applications. The institutional setting for KEI is founded on the regulatory regime, rule of law, population, and tariff and non-tariff barriers as indicators of openness. Other variables such as political stability, corruption control, and government effectiveness are important, but these are highly correlated such that there is no huge loss from excluding these in the creation of KEI. Finally, the independent variables are $y(t),{ỹ}_{i}(t),$ *and t* are as previously defined.

**Table 1:** Developing and emerging market economies, 1995–2017.

Algeria	CAR	Greece	Malawi	Pakistan	Sierra Leone
Angola	Chad	Guinea	Malaysia	Peru	S. Africa
Argentina	Colombia	Hungary	Mali	Philippines	Sudan
Benin	Comoros	India	Mauritania	Poland	Tanzania
Botswana	Czech Rep.	Indonesia	Mauritius	Qatar	Togo
Brazil	Djibouti	Israel	Mexico	Russia	Tunisia
Burkina Faso	Eq. Guinea	Kenya	Morocco	Rwanda	Turkey
Burundi	Eritrea	Lesotho	Mozambique	Sao Tome & P.	Uganda
Cabo Verde	Ethiopia	Liberia	Namibia	Saudi Arabia	UAE
China	Gabon	Libya	Niger	Senegal	Zambia
Cameroon	Ghana	Madagascar	Nigeria	Seychelles	Zimbabwe

Source: Own creation.

### 3.2 Methodology

Eq 1 is our generalized model the special cases of which are (2)–(7), alternatively the specific laws. For empirical estimations, we assume the logarithms of (7) in whole and in part as (2)–(6) separately, i.e.:

$$A_{i}(t)=\alpha_{0}+\alpha_{1}A_{i}(t-1)+\sum_{i}^{n}\alpha_{2}y_{i}(t)+\sum_{i}^{n}\alpha_{3}z_{i}(t)+\alpha_{4}t+\varepsilon_{i}(t),$$ (8)

where ${A_{i}(t)\equiv KEI_{i}(t),\alpha }_{0}={log(A}_{0}(t)),\varepsilon_{i}(t)=random\;error\;term,\alpha_{2}=w>0,\alpha_{3}=g>0,{z_{i}(t)=[{ỹ}_{i}(t)-y_{i}(t)],\alpha }_{4}=m>0,\varepsilon_{it}=\rho_{i}\varepsilon_{i}(t-1)+v_{i}(t)$.

## 4. Results and discussion

In the main results obtained for different laws of technological progress we focus on standardized coefficients and their associated p-values to stress the economic importance of the variables rather than their statistical significance [60]. In Table 2 the technological knowledge progress of DEMEs evolves according to Moore’s Law. They suggest that while the history of technological knowledge, *A*_*i*_(*t*−1), has delayed current progress, the results show that time (years) has been of crucial importance in these countries, contributing up to 99% towards progress for each one standard deviation. In Table 3, we assume that the motion of technological knowledge progress is according to Wright’s Law. In this case, the effects of cumulative GDP per capita is negative, i.e. $\frac{dlnA_{i}(t)}{dln{ỹ}_{i}(t)}<0$. This means that at the current average rate of cumulative learning, it will take 17.6 years for technological knowledge to double in this group of countries. However, according to Goddard’s Law if these countries sustain an annual rate of 14.1%, they will be able to double their technological knowledge progress in only 5.1 years (Table 4).

**Table 2:** Moore’s Law and technological progress in developing and emerging economies.

Variable	Standardized Coefficient (p-value)	Standardized Coefficient (p-value)
Log *A*_0_(*t*))	0.000 (0.000)	0.000 (0.000)
Lag (Log (*A*_*i*_(*t*)))		-0.007 (0.405)
Time (years)	0.9865 (0.000)	0.9865 (0.000)
LM, χ^2^ with 65 df (p-value)	678.15 (0.000)	670.16 (0.000)
Breusch-Pagan, χ^2^ with 2145 df (p-value)	46102.20 (0.000)	46044.60
F from Mean (p-value)	54976.317 (0.000)	27526.366 (0.000)
LLF	-11324.2	-11322.7
R-squared	0.9732	0.9732
Durbin H test statistic	N/A	40.302
Pooled Observations	1518	1452

Source: Own creation. Notes: LM: Test for cross-section heteroscedasticity. Breusch-Pagan: Test for diagonal covariance matrix. LLF: Log of the likelihood function.

**Table 3:** Wright’s Law and technological progress in developing and emerging economies.

Variable	Standardized Coefficient (p-value)	Standardized Coefficient (p-value)
Log (*A*_0_(*t*))	0.000 (0.308)	0.000 (0.298)
Lag (Log (*A*_*i*_(*t*)))		-0.002 (0.991)
Log (*y*_*it*_)	-0.041 (0.307)	-0.041 (0.296)
LM, χ^2^ with 65 df (p-value)	49114 (0.000)	449113 (0.000)
Breusch-Pagan, χ^2^ with 2145 df (p-value)	46923 (0.000)	46924 (0.000)
F from Mean (p-value)	2.538 (0.111)	1.272 (0.280)
LLF	-14069.0	-14069.0
R-squared	0.0017	0.0017
Durbin- H test statistic	N/A	N/A
Pooled Observations	1518	1452

Source: Own creation. Notes: LM: Test for cross-section heteroscedasticity. Breusch-Pagan: Test for diagonal covariance matrix. LLF: Log of the likelihood function.

**Table 4:** Goddard’s Law and technological progress in developing and emerging economies.

Variable	Standardized Coefficient (p-value)	Standardized Coefficient (p-value)
Log (*A*_0_(*t*))	0.000 (0.989)	0.000 (0.9890)
Lag (Log (*A*_*i*_(*t*)))		0.000 (1.000)
Log (*y*_*i*_(*t*))	0.1406 (0.000)	0.1406 (0.000)
LM, χ^2^ with 65 df (p-value)	47316 (0.000)	47316 (0.000)
Breusch-Pagan, χ^2^ with 2145 df (p-value)	24846 (0.000)	24851 (0.000)
F from Mean (p-value)	30.578 (0.000)	15.279 (0.000)
LLF	-14055.1	-14055.1
R-squared	0.0198	0.0198
Durbin- H test statistic	N/A	N/A
Pooled Observations	1518	1452

Source: Own creation. LM: Test for cross-section heteroscedasticity. Breusch-Pagan: Test for diagonal covariance matrix. LLF: Log of the likelihood function.

The results in Table 5 come from a combination of Wright’s Law and Moore’s Law, or what NFBT has called the Nordhaus’s Law. Here much progress comes from exogenous sources, but $\frac{\partial lnA_{i}(t)}{\partial {ỹ}_{i}(t)}$ = 0.156 as well. The latter implies that all else held constant this group can double its technological knowledge progress in 4.6 years. However, if we only consider *y*_*i*_(*t*) instead of ${ỹ}_{i}(t),$ the time factor contributes about 73% toward the doubling time. The small effect suggests that *it will take up to 131 years for this group to double* its current rate of progress (Table 6). This seems too dramatic; what it means is that the *in-situ* scale is very insufficient and too far from the frontier. The SKC’s Law predicts that on average these 66 countries can double their progress in 5.2 years if they keep the estimated scale at 13.87%, but if the cumulative learning rate stays at 3.5% the doubling will happen in 21 years (Table 7). The findings make sense [61]; point out that economic production scale can inhibit demand and hence technological progress [62].

**Table 5:** Nordhaus’s Law and technological progress in developing and emerging economies.

Variable	Standardized Coefficient (p-value)	Standardized Coefficient (p-value)
Log (*A*_0_(*t*))	0.000 (0.000)	0.000 (0.000)
Lag (Log ((*t*)))		-0.001 (0.317)
Log (*y*_*i*_(*t*))	0.1560 (0.000)	0.1560 (0.000)
Time (years)	1.0167 (0.000)	1.0167 (0.000)
LM, χ^2^ with 65 df (p-value)	771.80 (0.000)	772.88 (0.000)
Breusch-Pagan, χ^2^ with 2145 df (p-value)	38978 (0.000)	38941 (0.000)
F from Mean (p-value)	221431.790 (0.000)	27526.366 (0.000)
LLF	-9758.2	-9757.92
R-squared	0.9966	0.9966
Durbin–H test statistic	N/A	38.861
Pooled Observations	1518	1452

Source: Own creation. Notes: Test for cross-section heteroscedasticity. Test for diagonal covariance matrix. LLF: Log of the likelihood function.

**Table 6:** Goddard-Moore’s Laws and technological progress in developing and emerging economies.

Variable	Standardized Coefficient (p-value)	Standardized Coefficient (p-value)
Log (*A*_0_(*t*))	0.000 (0.000)	0.000 (0.000)
Lag (Log (*A*_0_(*t*)))		-0.007 (0.404)
Log (*y*_*i*_(*t*))	-0.0055 (0.000)	-0.0055 (0.040)
Time (years)	0.9873 (0.000)	0.9874 (0.000)
LM, χ^2^ with 65 df (p-value)	685 (0.000)	676.95 (0.000)
Breusch-Pagan, χ^2^ with 2145 df (p-value)	4502 4(0.000)	44959 (0.000)
F from Mean (p-value)	27501.248 (0.000)	18360.005 (0.000)
LLF	-11323.4	-11321.8
R-square	0.9966	0.9966
Durbin–H test statistic	N/A	40.307
Pooled Observations	1518	1452

Source: Own creation. Notes: LM: Test for cross-section heteroscedasticity. Breusch-Pagan: Test for diagonal covariance matrix. LLF: Log of the likelihood function.

**Table 7:** SKC’s Law and technological progress in developing and emerging economies.

Variable	Standardized Coefficient (p-value)	Standardized Coefficient (p-value)
Log (*A*_0_(*t*))	0.000 (0.521)	0.000 (0.376)
Lag (Log (*A*_*i*_(*t*)))		-0.001 (0.994)
Log (*y*_*i*_(*t*))	0.1387 (0.000)	0.1387 (0.000)
Log (${ỹ}_{i}(t)-y_{i}(t))$	-0.0347 (0.376)	-0.0348 (0.365)
LM, χ^2^ with 65 df (p-value)	47203 (0.000)	47203 (0.000)
Breusch-Pagan, χ^2^ with 2145 df (p-value)	39464 (0.000)	39475 (0.000)
F from Mean (p-value)	16.22910.813 (0.000)	27526.366 (0.000)
LLF	-14054.2	-14054.2
R-squared	0.0210	0.0210
Durbin–H test statistic	N/A	38.861
Pooled Observations	1518	1452

Source: Own creation. Notes: LM: Test for cross-section heteroscedasticity. Breusch-Pagan: Test for diagonal covariance matrix. LLF: Log of the likelihood function.

The results from (7) are mixed (Table 8). Here time has the most overwhelming effect. Surprisingly, the rate of cumulative learning is only 0.2%, suggesting that at that rate *these countries would need more the 480 years to double their progress*, but only 4.62 years if they maintain the growth of their scale at 15.6% per annum.

**Table 8:** Generalized laws of technological progress in developing and emerging economies.

Variable	Standardized Coefficient (p-value)	Standardized Coefficient (p-value)
Log (*A*_0_(*t*))	0.000 (0.000)	0.000 (0.000)
Lag (Log (*A*_*i*_(*t*)))		-0.0012 (0.315)
Log (*y*_*i*_(*t*))	-0.0015 (0.365)	-0.0015 (0.363)
Log (${ỹ}_{i}(t)-y_{i}(t))$	0.1560 (0.000)	0.1560 (0.000)
Time (years)	1.0171 (0.000)	1.0171 (0.000)
LM, χ^2^ with 65 df (p-value)	767.12 (0.000)	768.22 (0.000)
Breusch-Pagan, χ^2^ with 2145 df (p-value)	39198 (0.000)	39162 (0.000)
F from Mean (p-value)	147911.488.790 (0.000)	110904.146 (0.000)
LLF	-9756.22	-97555.92
R-squared	0.9966	0.9966
Durbin–H test statistic	N/A	38.860
Pooled Observations	1518	1452

Source: Own creation. Notes Test for cross-section heteroscedasticity. Breusch-Pagan: Test for diagonal covariance matrix. LLF: Log of the likelihood function.

It is worth pointing out that in all these the constant terms log (*A*_0_(*t*)) and historical rates of progress *log* (*A*_*i*_(*t*−1)) have been negative. The exception has been Goddard’s law where both the constant term and the coefficients of *log* (*A*_*i*_(*t*−1)) are positive, but their very high p-values (0.9890 and 1.000 respectively) recommend rejecting the null and accepting the alternative hypothesis. Although their study is at the microeconomic level [63], observed a “puzzle” which they attribute its existence to “nonlinearities” of “technological innovations” due to “various types of investment” (p. 1), see [62]. Table 9 summarizes the results, and these are likely influenced by the different investment decisions these countries made in technological progress locally [see, 64]. Moreover, our measure of technological knowledge, KEI, stands on four pillars: economic and institutional regime, human capital (education and skills), information and communication infrastructure, and national innovations systems. The pillars differ across countries and are affected differently by differ factors. [65] demonstrate that a city digital economy depends on technological capability (knowledge and the like) but these in turn are not independent of openness to international relations (trade, finance, migration, etc.) according to [66].

**Table 9:** Sources of progress and doubling times.

Table	Source of Progress	Coefficient	Doubling time by the Rule of 72	R-squared	Hypothesis- Decision
Moore’s Law	$\frac{dlnA_{i}(t)}{dt}$	0.9865		0.9732	Accept
Wright’s Law	$\frac{dlnA_{i}(t)}{dln{ỹ}_{i}(t)}$	-0.041	17.60	0.0017	Accept
Goddard’s Law	$\frac{dlnA_{i}(t)}{dlny_{i}(t)}$	0.141	5.12	0.0198	Accept
Nordhaus’s Law	$\frac{dlnA_{i}(t)}{dln{ỹ}_{i}(t)}$	0.156	4.62	0.9966	Accept
	$\frac{dlnA_{i}(t)}{dt}$	1.0167			
Goddard-Moore’s Law	$\frac{dlnA_{i}(t)}{dlny_{i}(t)}$	-0.0055	131	0.9966	Don’t Accept
	$\frac{dlnA_{i}(t)}{dt}$	0.9873			
Sinclair-Klepper-Cohen Law	$\frac{dlnA_{i}(t)}{dln{(ỹ}_{i}(t)-y_{i}(t))}$ $\frac{dlnA_{i}(t)}{dlny_{i}(t)}$	-0.0347 0.1387	20.75 5.19	0.0210	Accept
Generalized Laws	$\frac{dlnA_{i}(t)}{dlny_{i}(t)}$ $\frac{dlnA_{i}(t)}{\left(dln{ỹ}_{i}(t)-y_{i}(t)\right)}$ $\frac{dlnA_{i}(t)}{dt}$	-0.0015 0.1560 1.0171	480 4.62	0.9966	Don’t Accept

Source: Own creation.

To sum: This study sheds light on whether well-known laws of technological progress familiar to electronics and manufacturing engineering apply to the progress of technological knowledge in a cross-section panel of 66 DEMEs over the 1995–2017 period. The goal is to *assess whether these models are of any practical utility for predicting technological progress in these economies at the macroeconomic level as they do at the microeconomic level*. Estimation yielded mixed evidence. In Table 9 one can see that unlike in NFBT [36, p. 6] where “… most of the methods are quite similar in their performance”, here Moore’s Law and a hybrid of Moore’s Law confirm a positive relationship between technological knowledge progress and the progress of time. However, Wright’s Law indicates that the slow rate of learning delays these countries’ doubling time by 18 years. Similarly, but of opposite sign, Goddard’s Law predicts that this group of countries will double their rate of progress in a little over 5 years. This result is consistent with the prediction of the Nordhaus’s Law—which puts the doubling time at 4.6 years.

For the SKC’s Law the current scale (per capita GDP) allows for the doubling time in 5.2 years, but net cumulative learning delays doubling time by 21 years. Our own generalized formulation suggests that the log difference between cumulative gross domestic product (GDP) per capita (learning rate) and GDP per capita (*in-situ* scale) allows for the doubling in 4.62 years. Ignoring the arithmetic signs Wright’s Law, Goddard’s Law, and SKC’s Law make reasonable predictions although for different reasons. On the other hand, they have the lowest explanatory power (R-square). The inconsistency between the explanatory power and predictive power of the estimate cautions bold interpretations. Furthermore, the results of the combined Goddard-Moore, and our own generalization recommend that we “do not accept” the hypothesis that in-situ scale explains the technological knowledge progress in these countries.

The highlights of this study are the following:

- The role of technology and technological change in human progress is indisputable, but how technological knowledge progresses remains an open research topic.
- The goal of this short paper is to assess whether well-known laws of technological progress common to production engineering are of any practical utility for predicting technological knowledge progress in a cross-section panel of 66 developing and emerging market economies over the 1995–2017 period.
- We generalize the famous laws, and we find mixed evidence, with some laws explaining technological progress in our sample reasonably well while other appear to be off target.
- This evidence suggests that existing (in-situ) production capacity of this group of countries is too small such that it slows the rate of learning and lengthens the doubling time.

## 5. Conclusion

We can conclude that the laws of progress do not all give a unique solution in this case. Moore’s Law indicates a positive correlation between progress and time, 73%-100% probability for each standard deviation, making the passage of time the most important determinant of technological knowledge progress. The problem of a small *in-situ* scale translates into low cumulative GDP per capita, alternatively a slow learning rate. Originally, Moore predicted technology (represented by a chip) to change every 12–24 years but [67] have estimated 9.5 years. This is one reason why, we believe, [48, p. 30–9] has wondered whether cumulative output is a good measure of learning after all. In addition, learning must be adjusted for forgetting as well as quality, so that there is slow gross learning and even slower net learning [68–71]. These limitations point in the direction of future research. Even so, there is some supporting evidence that at least some of the laws of technological progress apply at the macroeconomic level in DEMEs. Consequently, we conclude that as a group these countries should learn how to progress. A weakness of this study is that we have no recommendations on how precisely individual countries can learn to progress. Even so, the evidence generally recommends that these countries raise their GDP per capita to support cumulative learning and hence technological knowledge progress. This recommendation is strongest for the African countries since the make up 67% of the sample.
